# Successful Aging Among Older LGBTQIA+ People: Future Research and Implications

**DOI:** 10.3389/fpsyt.2021.756649

**Published:** 2021-10-25

**Authors:** Henrique Pereira, Debanjan Banerjee

**Affiliations:** ^1^Faculty of Social and Human Sciences, Department of Psychology and Education, University of Beira Interior, Pólo IV, Covilha, Portugal; ^2^Research Centre in Sports Sciences, Health Sciences and Human Development (CIDESD), Vila Real, Portugal; ^3^Private Practitioner, Kolkata, India

**Keywords:** healthy aging, discrimination, old age, sexual minorities, LGBTQIA+

## Introduction

As the world population continues to increase, so does the number of older people. According to the United Nations' World Population Aging report (1), one in five people will be over 65 by the year 2050, providing greater visibility of the diversity among older people, particularly with respect to sexual orientation and gender identity. For instance, more than 39 million people in the U.S. are age 65 years or older including 2.4 million people who identify as lesbian, gay bisexual, transgender, queer, intersex, asexual and other (LGBTQIA+) (2). Since older people represent a growing demographic group, new and important challenges around successful and dignified aging arise for those who belong to gender and or sexual minority groups.

As more inclusive policies are enacted in countries around the world to address the social and legal needs of older LGBTQIA+ people (3), the reality of this diverse group of people will also become better known, including stress factors due to the intersections of ageism, homophobia, biphobia, transphobia, racism, or poverty (4). Therefore, older LGBTQIA+ people are subject to unique stressors associated with their minority status, and may face double discrimination due to their age and their LGBTQIA+ identity, making them more likely to experience health disparities (5).

## LGBTQIA+ Older People: Who Are They?

In many countries around the world, older LGBTQIA+ people grew up in a time when their sexual orientation and gender identity were considered mental illnesses and their sexual activities criminal or sinful acts. Being heterosexual and cisgender were the only valid identity labels accepted and, consequently, LGBTQIA+ people were invisible, marginalized, socially excluded, and discriminated against (6).

Before the human rights movements of the 1970's, and the Stonewall riots, laws did not acknowledge spouses and/or partners, healthcare services and healthcare access was hindered by the fear of discrimination, and most people were less likely to self-identify or disclose their identities. This invisibility created many obstacles when receiving formal care and kept LGBTQIA+ identities hidden from scientific research, making it difficult to obtain accurate data for this population. Past rejection by family members and friends may have enhanced the impacts of sexual stigma and adversity, forcing them to face social isolation, emotional distress, and health related problems. Notwithstanding the challenging and threatening start to their lives, many older LGBTQIA+ people now live comfortable with their sexual identities and display a manifest resilience to the stresses they felt earlier in their lives (7).

Factors such social isolation, difficulties in accessing health care, lack of social and family support, greater probability of not having children, and greater probability of having had lifetime exposure to experiences of discrimination and social stigma related to sexual and gender identity status (8) are all associated with the presence of negative health outcomes (9, 10). This reality is congruent with the Minority Stress Theory (10), which states that living with stressors throughout one's life negatively impacts on the health, well-being, and successful aging outcomes of older LGBTQIA+ people.

When compared to older heterosexual and cisgender people, older LGBTQIA+ people systematically show worse mental health outcomes (11); namely, more depression and anxiety (12–14), more emotional distress (15), less sexual and relationship satisfaction (16), more loneliness (17, 18), and higher risk of suicide (19). Associated with these disparities may be factors that accentuate vulnerability, such as living with Human Immunodeficiency Virus (HIV) infection (20, 21) and stigma internalization (22, 23). All these factors make the mental health needs of older LGBTQIA+ people an important concern, while creating the need to look for specific strategies to minimize these risks and, simultaneously, promote their successful aging.

## What Is Successful Aging for LGBTQIA+ Older People?

There is not a consensus regarding the definition of successful aging. However, we could define it as the ability of older people to manage the specific challenges of this stage of life in a prosperous and satisfactory way, with good functional, physical, and cognitive capacity, and an active involvement in psychosocial life (24). This definition contrasts with the notion of aging as pathology (25), which is centered on deficits and losses.

To adjust this definition further for older LGBTQIA+ people, we should include criteria that transcend the merely biomedical; namely, subjective criteria based on the experiences of older LGBTQIA+ people (26). Here, the following factors are usually included: mental health variables, coping strategies, social relationships, attitudes, emotional well-being, community involvement and continuous learning (27), resilience around negative social constructions of LGBTQIA+ identities (28), and the constant challenges associated with the aging process and successive coming-outs (29).

Many older LGBTQIA+ people may have developed strategies to deal with adversity at younger ages that prove useful in the latter parts of their lives (30, 31), helping them develop coping strategies that are potentially generalizable to other developmental tasks involved in the aging process. Thus, in turn, can provide psychological benefits for individuals (32). Many of these tasks involve a proactive response to various adverse situations and hostile environments (33, 34), usually involving LGBTQIA+-phobia and ageism.

In this sense, several protective factors have been identified in developing resilience against marginalization and heteronormativity (35) among older LGBTQIA+ people, including high levels of self-esteem and self-efficacy (36) [associated with a higher quality of life experience (37)], mastery (30), and hope (38) (associated with better mental health). On the other hand, maintaining the ability to bounce back from adverse situations and respond to hostile environments—and, consequently, reduce the risk of vulnerability in more advanced stages of the life cycle—for many older LGBTQIA+ people involves the development of families of choice, as well as community or underground groups (39, 40). The goal of these associations is obtaining social support and the reinterpretation of the processes of normalization of life sequences not in compliance with heteronormativity, as well as the possibility of redefining success in their life projects as a valid LGBTQIA+ person (41).

## Future Research: Potential Areas and Strategies

It is clear that older LGBTQIA+ people face specific challenges that their heterosexual and cisgender peers do not. Hence, it is important to develop community-based studies that provide more information on the particular challenges they face in navigating the aging process. Thus, we present a list of topics that could be explored in order to deepen knowledge about successful aging among older LGBTQIA+ people, from a global perspective:

### Health Care and Health Care Access

Assessing the quality of health care, the impact of experiences of discrimination on access to physical and mental health care, as well as the protective and positive factors that can facilitate health experiences of older LGBTQIA+ people, will be vital to promoting their successful aging. Although there is already an important body of research investigating accessing physical and mental healthcare, there are gaps in the pre-existing research, such as service, policy and research innovations that should further expand the knowledge in this field.

### Caregiving

Given that older LGBTQIA+ people often depend on families of choice to access social support and sometimes there are no structured support networks at all, it is important to assess what particular obstacles they face when receiving/seeking care, because the invisibility present in scientific research perpetuates this gap. At the same time, support structures and health professionals working with older LGBTQIA+ people often ignore their gender and sexual identities, forcing them to go back into the closet and endure again the isolation and erasure of an identity that took them so long to form and maintain.

### Cultural/Affirmative Competence

Older LGBTQIA+ people are more reluctant to seek out elder care centers, home and meal support programs, or other community support services because of the anticipation of rejection. After decades of exposure to experiences of discrimination and stigmatization based on their sexual and gender identities, many older LGBTQIA+ people assume that they will not be welcome in these structures. To understand the dynamics of these processes is fundamental to optimizing competent interventions.

### Social Isolation and Loneliness

Maintaining social networks and sources of social support is essential for successful aging, as it positively influences quality of life, physical and mental health, and happiness for older LGBTQIA+ people. Loneliness and isolation, by contrast, increase the risk of vulnerability, especially in older people. This vulnerable group may be at an enhanced risk due to prolonged minority stress and social ostracization.

### Wellness, Health Behaviors, and Quality of Life

Behaviors that promote well-being, such as not smoking, not drinking alcohol, practicing physical activity, or seeing the doctor regularly, are important factors in successful aging. Just as the minority stress experience significantly influences health disparities, creating an environment of well-being will reduce the risk of vulnerability.

### Impact of Discrimination

Many older LGBTQIA+ people have had very different generational experiences from those of contemporary LGBTQIA+ youth in most Western societies, where there is more openness and relatively safer spaces in which to express their sexual and gender identities. However, there are still many regions, countries and societies where these identities continue to be criminalized, either by laws or by social norms that force older LGBTQIA+ people to remain in the closet, with all the health costs associated with this isolation.

### HIV/AIDS

HIV impacts the LGBTQIA+ community, and older LGBTQIA+ people are no exception. The fact that HIV has become a chronic condition that allows people to live many more healthy years than in the 1980s and 1990s means there are many older LGBTQIA+ people who are HIV-positive, but who may experience the triple stigma of HIV, sexual minority status, and ageism.

### Independence/Loss of Decision Making

Given the likelihood of older LGBTQIA+ people having less social support, the maintenance of emotional and physical autonomy, as well as the ability to make life decisions about aging problems, such as economic issues, legal planning, end-of-life decisions, retirement decisions, among others, may represent important challenges in the search for answers free from judgment and prejudice.

### Spirituality, Religion, and Religiosity

For many older LGBTQIA+ people, spirituality and religion can serve as an important resource for maintaining peace and dignity at this stage of the life cycle. However, many religions are themselves centers of stigma and prejudice, leading many people to avoid religious institutions. Understanding how these dynamics may affect successful aging is fundamental.

### Life Course Trajectories

Many LGBTQIA+ people experience the need to carry out different developmental tasks throughout their lives, such as identity formation, stigma management, coming-out experiences, and adjusting to changes arising from family and social transformations, thus creating the need to assess the potential risk of the course of these trajectories.

### Lifetime Trauma

Examining the impact of traumatic and adverse experiences throughout the life cycle is an important task for studying the conditions of successful aging in LGBTQIA+ populations. Unfortunately, many people have gone through experiences of discrimination and victimization that could have severe consequences when it comes to maintaining their well-being, health and quality of life in general.

### Cultural Differences and Global Initiatives

Globalization and the widespread use of the internet have made it possible to understand the phenomena that affect the lives of older LGBTQIA+ people on a global scale. The different social structures in which they live represent a cultural challenge for research, but at the same time an opportunity for cross-cultural comparisons, which will inform how best to work with older populations in ways that are inclusive and affirmative, based on respect for diversity and human rights.

### COVID-19 Pandemic

The COVID-19 pandemic has meant many challenges for older people in general, due to their increased vulnerability to the disease, and for older LGBTQIA+ people in particular, due to the fact that lockdown measures imposed by most governments have accentuated pre-existing disparities; namely, social isolation, less advantageous health outcomes, discrimination in access to health care, or difficulties in managing complications associated with COVID-19. Research into their “dual vulnerability” during the pandemic is increasing. A recent exploration of the lived experiences of older transgender adults in India revealed the burden of ageism and gender/sexual identity, marginalization, lesser priority for healthcare, and multi-faceted existential threats during the lockdown (42). Spirituality and community rituals emerged as important resilience factors.

### Intersectional Approaches

The identity experience of older LGBTQIA+ people is diverse and multifactorial, often marked by experiences of marginalization. An intersectional approach to the study of their successful aging constitutes an opportunity to validate their sexual and gender identities while accommodating the rapid demographic, social and generational changes to which they are subject.

### Qualitative Studies

More qualitative studies that document the needs and vulnerabilities, but also the protective factors associated with the successful aging of older LGBTQIA+ people are needed, as this will allow us to dig deeper into the reality of their life experiences.

### Longitudinal and Population Studies

Carrying out longitudinal and population-based studies will allow for the assessment of vulnerability trajectories over time, as well as the identification of the groups most exposed to risk. Doing so will help to anticipate appropriate preventive interventions.

### Examining Different Contexts and Different Generations

The experience of LGBTQ+ people vary by context and time. LGBTQIA+ people living in a more progressive/friendly context will have very different experiences than their counterparts living in more restrictive contexts. The same principle is relevant for LGBTQIA+ people born in the 1950s and those born in the 1980s. For instance, the New Gay Teenager hypothesis claims that LGBTQIA+ youth today are different from previous cohorts and are more like other youth, regardless of sexual orientation (43). In contrast, the aging thesis claims that LGBTQIA+ youth today face experiences similar to their counterparts in the past, and thus they are still considered to be high-risk youth with special needs in terms of social services (44). Therefore, examining differences between age-groups within the framework of aging is necessary to better understand the successful aging process.

### Innovative Studies

Future studies that may be developed regarding successful aging with older sexual minorities need to offer methodological responses based on the incorporation of measures sensitive to the diverse nature of older LGBTQIA+ people, focused on the ability to achieve hidden-within-hidden populations.

Despite enormous social and scientific advances in LGBTQIA+ aging, most people remain invisible when it comes to areas of intervention, services, policy, and formal research. The articulation of the topics that we propose here with the creation of visibility and validation of older LGBTQIA+ people will allow us to create structural changes that will positively affect their lives, involving community partners who will inform us about their strengths and vulnerabilities. Working on this strategy will allow the creation of affirmative and culturally responsible programs and policies, directly contributing to the elimination of invisibility and, as such, with direct implication in psychogeriatrics, promoting equality and a sense of belonging, which are fundamental for successful aging.

## Research Implications in Psychogeriatrics

Until a few decades ago, the aging of LGBTQIA+ people was completely invisible to researchers. Despite much being done, and despite the population's rapid growth, they remain invisible in many segments of society, including in aging services, policies, and research (45). Therefore, we present a set of important implications for directly promoting the successful aging of older sexual minorities:

### Intervention

Models of successful aging among older LGBTQIA+ people should incorporate the identification of modifiable factors to promote people's well-being, integrating different possible configurations in the responses to the specific needs assessed. In this sense, it is useful that interventions can be based on an aging perspective that emphasizes critical reflexivity, allowing micro and macro views of structural issues that may be interfering with interventions; namely, heteronormativity, heterosexism, homophobia, biphobia or transphobia (46). With this, professionals working with older LGBTQIA+ people will be better informed about the social dynamics that will allow them to intervene more effectively in key areas such as: the effects of stigma and prejudice on physical and mental health; working with families of origin and of choice; the management of difficulties associated with legal inequalities that may exist; overcoming existing barriers to health care and eliminating or reducing inequalities; management of formal psychosocial support structures; and offering responses focused on alleviating specific problems for older LGBTQIA+ people, among others.

### Theoretical Work

Theoretical construction in the field of LGBTQIA+ aging must be aware of the current socio-historical contexts, ready to offer conceptual contributions and provide explanatory models that accommodate a broad and intersectional view of what it means to age successfully for older LGBTQIA+ people (47). It is likely that minority stress models or resilience models will not be enough to support investigations and should be complemented by critical gerontology models or health equity promotion models. This means that existing social structures must be challenged to recognize the psychosocial processes of aging, reject heteronormativity, and offer validity and attention to the discourses of the most fragile LGBTQIA+ identities, such as bisexual, transgender, or queer identities.

### Social Policy and Legislation

Older LGBTQIA+ people need legal protection from discrimination based on their sexual gender identity status, including access to physical, mental, and occupational health care, living arrangements, and eliminating disparities (48). In this sense, it is necessary for governments and policymakers to incorporate the needs of older LGBTQIA+ people in their political agenda. These decisions must be informed by competent investigations about their real needs, as well as by partnerships with community organizations working to advance the rights of LGBTQIA+ people, based on the principles of active participation and social change.

### Promoting Visibility

From a life cycle perspective, the aging of LGBTQIA+ people is often accompanied by the existence of hurdles associated with their sexual and gender identity status, creating invisibility and often causing older LGBTQIA+ people to return to the closet at this stage of life (49). This movement represents a setback in life trajectories and is usually accompanied by feelings of anticipation of rejection (especially in a residential context); it is also associated with the impossibility of freely expressing one's sexual and/or gender identities, thus losing opportunities to offer care and support adjusted to their needs. It is therefore critical to challenge invisibility and allow older LGBTQIA+ people to voice their needs as well as have more positive appraisal of their identities in daily life.

### Expanding Knowledge

It is necessary to use the strategy of fighting for the rights of older LGBTQIA+ people through the research that is produced. It is crucial to obtain reliable data that will help arrive at a greater and deeper understanding of their needs, as this will allow us to offer tailored and more effective responses. On the other hand, it will be convenient to create local, regional, and national forums to discuss these needs, as this will allow for broader visions of what is really needed and, in this way, create opportunities to expand this knowledge to mass media and educational spaces throughout society.

### Creating Effective LGBTQIA+ Aging Infrastructures

Improving the aging process of older LGBTQIA+ people is a complex challenge with many important tasks. To offer opportunities for improvement, the existence of effective infrastructures is essential, from organizations that promote the rights of older LGBTQIA+ people to the possibility of offering specific structures for people that accommodate their needs. Many older LGBTQIA+ people may not want a specific home just for themselves, but most likely prefer to be in a home where their needs as an LGBTQIA+ person are respected and valued (50). This will also promote social cohesion and social connectedness in this community.

### Access to Education/Training Opportunities

Professionals who work with older people and are involved in the task of offering competent and adequate care services to their clients should actively seek training programs at the university level, specifically targeted at aging and LGBTQIA+ topics, in each of their areas of intervention, whether in the field of physical, mental, or social health (51). With this, they will be able to improve their skills, adjust services and directly contribute to the successful aging of older LGBTQIA+ people (52).

### Human Dignity

A human rights framework based on human dignity is critical to bringing about change in promoting the well-being of older LGBTQIA+ people. The dignity of older people is a multidimensional construct that involves self-respect, social acknowledgment, independence, and privacy (53), but which can be negatively affected by ageism, homophobia, biphobia, or transphobia. For this reason, an approach based on human dignity will be fundamental.

The knowledge generated by researching the aging experiences of LGBTQIA+ people should be disseminated by all agencies working with older people, guiding the implementation of future investigations without omitting sexual and gender identities. This approach is essential for creating visibility, but also for recommending best intervention practices. Older LGBTQIA+ people represent a heterogeneous group of people, and this expansion of knowledge will allow an understanding of the mechanisms inherent to the trajectories of subgroups within the LGBTQIA+ community, offering safer clues for the promotion of successful aging.

## The Way Forward

Older LGBTQIA+ people represent a diverse group of people who are still exposed to adversity, stigma, marginalization, and discrimination, with a greater probability of isolation, less social support, and therefore more risk of having worse physical, mental, and social health indicators. Heteronormative aging models do not adapt to the specific needs of older LGBTQIA+ people and are marked by a double stigmatization lens (LGBTQIA+-phobia and agism). The result is a thick invisibility, which is incompatible with the creation of formal and informal environments that promote successful aging and the fight against loneliness and social isolation. By conducting studies based on the investigation of the needs and lived experiences of older LGBTQIA+ people that integrate critical and adjusted perspectives, it will be possible to more effectively address the risk and protective factors that older LGBTQIA+ populations face around the world.

The application of multi-level resilience models should also be very helpful in future research on successful aging, since LGBTQIA+ older adults experience multiple stigmatizations and consistently show health disparities (54). Consequently, informal caregiving may play an important role in successful aging within the LGBTQIA+ community, and affirmative key competencies should be developed for working with this population.

Despite the risks and vulnerabilities that older LGBTQIA+ people experience, positive health outcomes in later life are also possible, especially resilient pathways where psychological resources (e.g., positive identity appraisal) and social resources (e.g., social connectedness) are associated with health-promoting behaviors, which in turn facilitate good overall health into older age (55). These findings suggest that the interaction of social and psychological factors can help LGBTQIA+ older adults to maintain good health and foster successful aging, even within an environmental context of marginalization (56). Therefore, successful aging is possible in older LGBTQIA+ individuals, as psychological and social resilience resources may compensate for the impact of disadvantage.

## Author Contributions

All authors listed have made a substantial, direct and intellectual contribution to the work, and approved it for publication.

## Conflict of Interest

The authors declare that the research was conducted in the absence of any commercial or financial relationships that could be construed as a potential conflict of interest.

## Publisher's Note

All claims expressed in this article are solely those of the authors and do not necessarily represent those of their affiliated organizations, or those of the publisher, the editors and the reviewers. Any product that may be evaluated in this article, or claim that may be made by its manufacturer, is not guaranteed or endorsed by the publisher.

## References

[1] United Nations. World Population Ageing. (2019). Department of Economic and Social Affairs Population Division. United Nations: New York. Available online at: https://www.un.org/en/development/desa/population/publications/pdf/ageing/WorldPopulationAgeing2019-Highlights.pdf (accessed August 3, 2021).

[2] American Psychological Association. Lesbian, Gay, Bisexual and Transgender Aging. Available online at: https://www.apa.org/pi/lgbt/resources/aging (accessed Sep 28, 2021).

[3] OECD. Over the Rainbow? The Road to LGBTI Inclusion. Paris: OECD Publishing (2020).

[4] Fredriksen-Goldsen KI Jen S Muraco A. Iridescent life course: LGBTQ aging research and blueprint for the future – a systematic review. Gerontology. (2019) 65:253–74. 10.1159/000493559 30826811 PMC6779307

[5] Pereira H. The impacts of sexual stigma on the mental health of older sexual minority men. Aging Mental Health. (2021). [Epub ahead of print]. 10.1080/13607863.2021.1916883 33949273

[6] Abatiell P Adams M. LGBT aging: a question of identity. Gerontologist. (2011) 51:880–4. 10.1093/geront/gnr113

[7] Bower KL Lewis DC Bermúdez JM Singh A. Narratives of generativity and resilience among LGBT older adults: leaving positive legacies despite social stigma and collective trauma. J Homosexuality. (2021) 68:230–51. 10.1080/00918369.2019.1648082 31407964

[8] SAGE and the National Resource Center on LGBT Aging. Facts on LGBT Aging. Available online at: https://www.sageusa.org/wp-content/uploads/2021/05/sage-lgbt-aging-final-2021.pdf (accessed August 3, 2021).

[9] Fredriksen-Goldsen KI Kim H-J Emlet CA Muraco A Erosheva EA Hoy-Ellis CP. The Aging and Health Report: Disparities and Resilience among Lesbian, Gay, Bisexual, and Transgender Older Adults - Key Findings Fact Sheet. Seattle, WA: Institute for Multigenerational Health (2011).

[10] Meyer IH. Prejudice, social stress, and mental health in lesbian, gay, and bisexual populations: conceptual issues and research evidence. Psychol Bull. (2003) 129:674–97. 10.1037/0033-2909.129.5.674 12956539 PMC2072932

[11] Semlyen J King M Varney J Hagger-Johnson G. Sexual orientation and symptoms of common mental disorder or low wellbeing: combined meta-analysis of 12 UK population health surveys. BMC Psychiatry. (2016) 16:1–9. 10.1186/s12888-016-0767-z 27009565 PMC4806482

[12] Bostwick WB Boyd CJ Hughes TL West BT McCabe SE. Discrimination and mental health among lesbian, gay, and bisexual adults in the United States. Am J Orthopsychiatry. (2014) 84:35–45. 10.1037/h0098851 24826824 PMC4144327

[13] Chakraborty A McManus S Brugha TS Bebbington P King M. Mental health of the non-heterosexual population of England. Br J Psychiatry. (2018) 198:143–8. 10.1192/bjp.bp.110.082271 21282785

[14] Pereira H de Vries B Serrano JP Afonso RM Esgalhado G Monteiro S. Depression and quality of life in older gay and bisexual men in Spain and Portugal. Int J Aging Hum Dev. (2020) 91:198–213. 10.1177/0091415019864600 31339330

[15] Ribeiro-Gonçalves JA Costa PA Leal I. Psychological distress in older Portuguese gay and bisexual men: the mediating role of LGBT community connectedness. Int J Sexual Health. (2019) 31:407–13. 10.1080/19317611.2019.1670315

[16] Ribeiro-Gonçalves JA Costa PA Leal I. Minority stress in older Portuguese gay and bisexual men and its impact on sexual and relationship satisfaction. Sexuality Res Soc Policy. (2020) 17:209–18. 10.1007/s13178-019-00385-1

[17] Ribeiro-Gonçalves JA Pereira H Costa PA Leal I de Vries B. Loneliness, social support, and adjustment to aging in older Portuguese gay men. Sexuality Res Soc Policy. (2021). [Epub ahead of print]. 10.1007/s13178-021-00535-4

[18] Pereira H de Vries B Esgalhado G Serrano JP. Loneliness perceptions in older portuguese gay and bisexual men. J Homosex. (2021). [Epub ahead of print]. 10.1080/00918369.2021.1901504 33754962

[19] Li L. Suicide prevention in older adults: evidence-based approaches for care. Innovat Aging. (2020) 4:624–5. 10.1093/geroni/igaa057.2129

[20] Mazonson P Berko J Loo T Kane M Zolopa A Spinelli F. Loneliness among older adults living with HIV: the “older old” may be less lonely than the “younger old”. AIDS Care. (2021) 33:375–82. 10.1080/09540121.2020.1722311 32048520

[21] Batista IC Pereira H. Mental health, resilience and HIV in older gay and bisexual men. Educ Gerontol. (2020) 46:525–39. 10.1080/03601277.2020.1785673

[22] Barrett C Whyte C Comfort J Lyons A Crameri P. Social connec- tion, relationships and older lesbian and gay people. Sexual Relationship Ther. (2015) 30:131–42. 10.1080/14681994.2014.963983 25544830 PMC4270424

[23] Fredriksen-Goldsen KI Emlet CA Kim HJ Muraco A Erosheva EA Goldsen J. The physical and mental health of lesbian, gay male, and bisexual (LGB) older adults: the role of key health indicators and risk and protective factors. Gerontologist. (2013) 53:664–75. 10.1093/geront/gns123 23034470 PMC3709843

[24] Depp CA Jeste DV. Definitions and predictors of successful aging: a comprehensive review of larger quantitative studies. Am J Geriatr Psychiatry. (2006) 14:6–20. 10.1097/01.JGP.0000192501.03069.bc 16407577

[25] Zhang W Liu S Wu B. Defining successful aging: perceptions from elderly Chinese in Hawai'i. Gerontol Geriatr Med. (2018) 4:1–7. 10.1177/2333721418778182 30035192 PMC6050632

[26] Lee JE Kahana B Kahana E. Successful aging from the viewpoint of older adults: development of a Brief Successful Aging Inventory (SAI). Gerontology. (2017) 63:359–71. 10.1159/000455252 28297704

[27] Van Wagenen A Driskell J Bradford J. “I'm still raring to go”: successful aging among lesbian, gay, bisexual, and transgender older adults. J Aging Stud. (2013) 27:1–14. 10.1016/j.jaging.2012.09.001 23273552 PMC3534855

[28] Hash KM Rogers A. Clinical practice with older LGBT clients: overcoming lifelong stigma through strength and resilience. Clin Soc Work J. (2013) 41:249. 10.1007/s10615-013-0437-2

[29] Pereira H de Vries B Serzedelo A Serrane JP Afonso RM Esgalhado G. Growing older out of the closet: a descriptive study of older lgb persons living in Lisbon, Portugal. Int J Aging Hum Dev. (2019) 88:422–39. 10.1177/0091415019836107 30868915

[30] Wight RG LeBlanc AJ de Vries B Detels R. Stress and mental health among midlife and older gay-identified men. Am J Public Health. (2012) 102:503–10. 10.2105/AJPH.2011.300384 22390515 PMC3337756

[31] Brown LB Alley GR Sarosy S Quarto G Cook T. Gay men: aging well! J Gay Lesbian Soc Serv. (2001) 13:41–54. 10.1300/J041v13n04_06

[32] Orel NA. Gay, lesbian, and bisexual elders: expressed needs and concerns across focus groups. J Gerontol Soc Work. (2004) 43:57–77. 10.1300/J083v43n02_05

[33] Fredriksen-Goldsen KI Kim HJ Shiu C Goldsen J Emlet CA. Successful aging among LGBT older adults: physical and mental health-related quality of life by age group. Gerontologist. (2015) 55:154–68. 10.1093/geront/gnu081 25213483 PMC4542897

[34] Rowan NL Butler SS. Resilience in attaining and sustaining sobriety among older lesbians with alcoholism. J Gerontol Soc Work. (2014) 57:176–97. 10.1080/01634372.2013.859645 24797211

[35] Pilkey B. Queering heteronormativity at home: older gay Londoners and the negotiation of domestic materiality. Gend Place Cult. (2014) 21:1142–57. 10.1080/0966369X.2013.832659

[36] Witten TM. Elder transgender lesbians: exploring the intersection of age, lesbian sexual identity, and transgender identity. J Lesbian Stud. (2015) 19:73–89. 10.1080/10894160.2015.959876 25575324

[37] Emlet CA Fredriksen-Goldsen KI Kim HJ. Risk and protective factors associated with health-related quality of life among older gay and bisexual men living with HIV disease. Gerontologist. (2013) 53:963–72. 10.1093/geront/gns191 23355449 PMC3826162

[38] Sagie O. Predictors of well-being among older gays and lesbians. Soc Indic Res. (2015) 120:859–70. 10.1007/s11205-014-0608-8

[39] Neville S Kushner B Adams J. Coming out narratives of older gay men living in New Zealand. Australas J Ageing. (2015) 34(2 Suppl. 2):29–33 10.1111/ajag.12277 26525444

[40] Kong TS. A fading Tongzhi heterotopia: Hong Kong older gay men's use of spaces. Sexualities. (2012) 15:896–916. 10.1177/1363460712459308

[41] Fabbre VD. Gender transitions in later life: a queer perspective on successful aging. Gerontologist. (2015) 55:144–53. 10.1093/geront/gnu079 25161264 PMC4542896

[42] Banerjee D Rao TS. “The Graying Minority”: lived experiences and psychosocial challenges of older transgender adults during the COVID-19 pandemic in India, a qualitative exploration. Front Psychiatry. (2020) 11:604472. 10.3389/fpsyt.2020.604472 33488427 PMC7820119

[43] Savin-Williams RC. The New Gay Teenager. Cambridge, MA: Harvard University Press (2009).

[44] Pizmony-Levy O Shilo G Pinhasi B. Is there a new Israeli gay teenager? J LGBT Youth. (2009) 6:340–68. 10.1080/19361650903303514

[45] Fredriksen-Goldsen KI. The future of LGBT+ aging: a blueprint for action in services, policies, and research. Generations. (2016) 40:6–15. Available online at: https://www.ingentaconnect.com/content/asag/gen/2016/00000040/00000002/art00002 28366980 PMC5375167

[46] Fabbre V. Queer aging: implications for social work practice with lesbian, gay, bisexual, transgender, and queer older adults. Social Work. (2017) 62:73–6. 10.1093/sw/sww076 28395042

[47] Fabbre VD Jen S Fredriksen-Goldsen K. The State of Theory in LGBTQ aging: implications for gerontological scholarship. Res Aging. (2019) 41:495–518. 10.1177/0164027518822814 30626272 PMC6760910

[48] Redcay A McMahon S Hollinger V Mabry-Kourt HL Cook TB. Policy recommendations to improve the quality of life for LGBT older adults. J Hum Rights Soc Work. (2019) 4:267–74. 10.1007/s41134-019-00103-2

[49] Marhánková JH. Places of (in)visibility. LGB aging and the (im)possibilities of coming out to others. J Aging Stud. (2019) 48:9–16, 10.1016/j.jaging.2018.11.002 30832935

[50] Pereira H Serrano JP de Vries B Esgalhado G Afonso RM Monteiro S. Aging perceptions in older gay and bisexual men in Portugal: a qualitative study. Int J Aging Hum Dev. (2018) 87:5–32. 10.1177/0091415017720889 28748709

[51] Rogers A Rebbe R Gardella C Worlein M Chamberlin M. Older LGBT adult training panels: an opportunity to educate about issues faced by the older LGBT community. J Gerontol Soc Work. (2013) 56:580–95. 10.1080/01634372.2013.811710 23905835

[52] Higgins A Downes C Sheaf G Bus E Connell S Hafford-Letchfield T. Pedagogical principles and methods underpinning education of health and social care practitioners on experiences and needs of older LGBT+ people: findings from a systematic review. Nurse Educ Pract. (2019) 40:102625. 10.1016/j.nepr.2019.102625 31541934

[53] Banerjee D Rabheru K Lima CAM Ivbijaro G. Role of dignity in mental healthcare: impact on ageism and human rights of older persons. Am J Geriatric Psychiatry. (2021) 29:1000–8. 10.1016/j.jagp.2021.05.011 34167896

[54] Speh K von Humboldt S Leal I. LGBT in old age. In: Gu D Dupre M, editors. Encyclopedia of Gerontology and Population Aging. Springer (2019) Cham. 10.1007/978-3-319-69892-2_76-1

[55] Fredriksen-Goldsen KI Kim H Bryan AEB Shiu C Emlet CA. The cascading effects of marginalization and pathways of resilience in attaining good health among LGBT older adults. Gerontologist. (2017) 57:S72–83. 10.1093/geront/gnw170 28087797 PMC5241752

[56] Thoma MV McGee SL. Successful aging in individuals from less advantaged, marginalized, and stigmatized backgrounds. Clin Psychol Eur. (2019) 1:1–12. 10.32872/cpe.v1i3.32578

